# Mother–Young Bonding: Neurobiological Aspects and Maternal Biochemical Signaling in Altricial Domesticated Mammals

**DOI:** 10.3390/ani13030532

**Published:** 2023-02-02

**Authors:** Cécile Bienboire-Frosini, Míriam Marcet-Rius, Agustín Orihuela, Adriana Domínguez-Oliva, Patricia Mora-Medina, Adriana Olmos-Hernández, Alejandro Casas-Alvarado, Daniel Mota-Rojas

**Affiliations:** 1 Department of Molecular Biology and Chemical Communication, Research Institute in Semiochemistry and Applied Ethology (IRSEA), 84400 Apt, France; 2 Animal Behaviour and Welfare Department, Research Institute in Semiochemistry and Applied Ethology (IRSEA), 84400 Apt, France; 3 Facultad de Ciencias Agropecuarias, Universidad Autónoma del Estado de Morelos, Cuernavaca 62209, Mexico; 4 Neurophysiology, Behavior and Animal Welfare Assessment, DPAA, Universidad Autónoma Metropolitana, Xochimilco Campus, Mexico City 04960, Mexico; 5 Facultad de Estudios Superiores Cuautitlán, Universidad Nacional Autónoma de Mexico (UNAM), Cuautitlán Izcalli 54740, Mexico; 6 Division of Biotechnology—Bioterio and Experimental Surgery, Instituto Nacional de Rehabilitación-Luis Guillermo Ibarra Ibarra (INR-LGII), Tlalpan, Mexico City 14389, Mexico

**Keywords:** imprinting, bonding, maternal recognition, olfactory, maternal anogenital licking, vocalization

## Abstract

**Simple Summary:**

Mother–young bonding is an essential process that increases newborn survival through selective maternal care. In this mechanism, olfactory, visual, auditory, and tactile stimuli coming from the offspring activate specific brain structures to establish the affective recognition of the neonate. Immature species at birth, such as altricial animals, require extensive care, and their post-birth maturation influences the degree of maternal interaction. This review aims to discuss the neurobiological aspects of bonding processes in altricial mammals, with a focus on the brain structures and neurotransmitters involved and how these influence the signaling during the first days of the life of newborns.

**Abstract:**

Mother–young bonding is a type of early learning where the female and their newborn recognize each other through a series of neurobiological mechanisms and neurotransmitters that establish a behavioral preference for filial individuals. This process is essential to promote their welfare by providing maternal care, particularly in altricial species, animals that require extended parental care due to their limited neurodevelopment at birth. Olfactory, auditory, tactile, and visual stimuli trigger the neural integration of multimodal sensory and conditioned affective associations in mammals. This review aims to discuss the neurobiological aspects of bonding processes in altricial mammals, with a focus on the brain structures and neurotransmitters involved and how these influence the signaling during the first days of the life of newborns.

## 1. Introduction

Mother–young bonding refers to the early learning process by which the dam and offspring create a selective bond that allows both to identify each other, to enhance newborn survival, and to enable the acquisition of a preference for a particular type of stimulus [1,2,3]. At the time of parturition, the mother is the main source of protection, warmth, and initial postnatal feeding through lactation, as the young are unable to survive without milk, and adequate maternal care [4,5,6]. In addition, males and other filial members can also contribute [4,5].

The beneficial impact of maternal protection through social behaviors directed at newborns has been widely reported in animals, especially mammals [7,8,9,10,11,12,13,14]. The neural pathways activated to establish a mother–young preference [15] are important to avoid misdirected care, reduce energy outlays, and enhance reproductive success. The degree of interaction can also affect the offspring’s social behavior, notably maternal behavior, when reaching adulthood [16,17,18], and the effect that maternal behavior can have on the stress response of neonates [19]. Finally, maternal behavior and the interest of the newborn in his/her mother decreases when the offspring are mature enough to explore and feed themselves outside of the nest [20]. The relationship between the mother and the offspring is extremely important to promote the welfare of both.

This process of mutual recognition demands a brain that can be able to respond to multimodal sensory signals [15]. In this sense, the perception of different signals during bonding depends on the activation of several structures such as the locus coeruleus, or the olfactory, auditory, and visual cortices [21]. These areas interconnect with other regions to promote the secretion of neurotransmitters associated with the behavioral responses observed during this process, promoting maternal behaviors [22]. Significant evolution of the neocortex has been documented in monkeys and apes. This increased complexity is associated with the level of social relationships and strategies that serve to establish links between individuals that live in groups [23].

Another relevant issue associated with the requirement of protection and care of offspring is related to the maturity it has at parturition (precocial vs. altricial young; selective vs. non-selective bond) [24]. According to this, altricial species such as canids, rodents, marsupials, cricetids, and felines [25], are those that are more immature at birth, unable to move without help, require continuous parental protection during the first weeks of life, and must be groomed and provided with food [25]. Canine puppies, for example, are typically born with non-functional ears and eyes and are severely limited in regards to mechanical movement [26], so they depend on the dam and on their maternal behavior to survive. In this species, some mothers form a nest in a safe place where the young (born with an immature musculoskeletal system) are safe from predators [14]. Sows have the peculiarity that, although the newborn piglet has one of the highest motor and sensory developments, the mother builds a nest and the litter remains there for approximately one week [25].

Due to the influence that several brain structures, neurotransmitters, and the species have in the development of mother–young recognition, this review aims to analyze the neurobiological aspects of bonding processes. To achieve that, we will focus on the neurophysiological mechanisms of the mothers in mammal altricial species, including the brain structures, neurotransmitters, and communication channels involved.

## 2. Classification of Altricials, Precocials and Semialtricials

**Figure 1:**
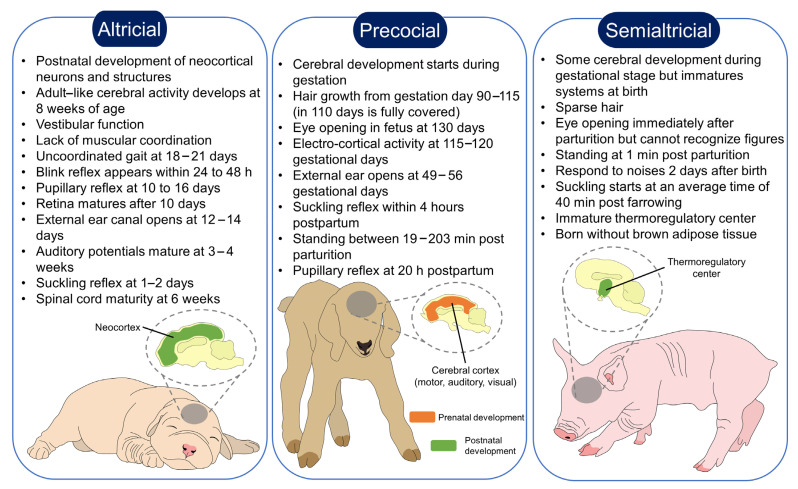
Neurodevelopmental differences between altricial, precocial, and semi-altricial species. According to the type of species, newborns from altricial, precocial, and semi-altricial females establish a different degree of bonding. In the case of precocial species, whose cerebral development starts during gestation, the time they require for maternal nursing is shorter since they can stand and move freely almost immediately after birth. In contrast, the maturation of sensorial systems in altricial species occurs during the postnatal period, where they are completely dependent on the mother to survive. Semi-altricial species are in between; while some of their senses are functional at birth, the mother nurses them until they are highly independent after several days after parturition.

Altricial and precocial species can be differentiated by the degree of the physical and behavioral developmental stage at birth (Figure 1) [25,27,28]. Altricial species (e.g., canids, felids, most rodents, and lagomorphs) are characterized by a lack of fur, impeding their adequate thermoregulation, and limited sensorial and musculoskeletal capabilities [24,29,30]. They must be fed and cared for over an extended period by their parents, and mothers tend to build a nest or seek a sheltered area to give birth to undeveloped offspring with closed eyes [14].

For example, rat pups reach postnatal ambulation by Day 10, and just after leaving the nest (approximately on Days 17–21), they increase their locomotor activity [31]. Generally, motor development is measured by the righting reflex, vibrissae placing, and righting reflex in rat pups, as well as eye-opening and spontaneous locomotor activity. The presentation of these reflexes in newborns is altered by exposure to stressful situations in the dams, showing full development 14 days post-parturition. This shows how the prenatal stage influences not only the response from the mother but the offspring as well [32]. To compensate for the delayed maturation, murine mothers spend approximately a total of 10.6% of their time in the first week of life grooming and licking the pups [33]. In the case of dogs, although the vestibular function is present since birth, puppies lack muscular coordination and exploratory movements; they start using their limbs 7–10 days after parturition [34]. Marsupials are another important example of altricial species since the young are born in an embryonic state and require to be carried by their mothers at all times [35].

In contrast, offspring from precocial species (e.g., bovines, goats, sheep, and equines) can feed themselves, and follow their mother after birth, which is associated with a rapid recognition process between the two [36]. They have a completely functional vision and hearing since birth, and a locomotor system that is sufficiently well-developed to allow them to stand effectively and start suckling [37]. They also have efficient thermoregulatory systems and may be quite independent at an early age [38,39]. In some cases, precocial species hide and do not follow their mothers; the mothers move approximately 100 m away from the young to graze and return to nurse them. As these young do not move, their energy requirements are minimal, so all the food they consume translates into rapid growth [40,41].

Semi-altricial and semi-precocial species, such as primates and pigs, are those whose young have certain mobility or sensory independence, but still require prolonged maternal care [28]. The semi-altricial species have functional sensorial systems (e.g., auditory and visual), but deficient locomotor and thermoregulation. In primates, newborns are taken care of in a communal rearing system [42]. In domestic swine, nest-building is a natural behavior of the species since the offspring remains inside the nest for the first two weeks after farrowing [24,43].

Between types of species, there are some reported differences such as brain size and cognition, which may particularly influence the bonding systems established by altricial animals [25]. It is said that maternal investment and litter size influences brain size [44] and, at birth, altricial species have smaller brains [45] since most of the cerebral development occurs at the same time as eye-opening [46]. However, although their brain sizes might be smaller at birth when compared to precocial animals, their growth rate is higher during the first day post-birth [45].

An important fact to mention is that maturity at birth could also be associated with the status of the species being prey or predator. For example, precocial animals that stand up immediately after birth can avoid predators and escape [47], while altricial neonates require maternal protection, making them an easy prey when parents are not near the nest [48]. These differences are also related to the ability of the mother and the young to recognize each other in a very limited period where the brain and neuroendocrine pathways can form the bonding. Table 1 compares key factors associated to maternal–fetal bonding in altricial and precocial species.

## 3. Sensitive Period

The process of filial bonding between the mother and the neonate is triggered during a decisive bounded interval called the sensitive (or critical) period [56,57,58]. In most animals, this period occurs during the first 4 to 12 h post-parturition [59]. Failure of maternal recognition can be due to interruptions caused by other animals or by human interventions. When these interruptions happen, reproductive, social, or behavioral disturbances, such as the mother’s rejection of her offspring, can occur [60]. Specifically, in rats, odor-preference learning is achieved in the first 10 postnatal days and requires low levels of corticosterone in pups to maintain the normal length of the period [61]. In the case of rabbits, the first post-partum week is considered the sensitive period for pups to react to the mother or human intervention [62].

In the case of dogs, a sensitive period is considered to be established during the first two weeks of life, when they are highly dependent on their mother, and it is known that alteration in the maternal care or attachment during this period can negatively influence the behavior in adult dogs [63]. For kittens, a similar amount of time is considered the critical period, and during this time the mother is dedicated to nursing and feeding the newborns, only spending time away from the newborns when they become three weeks old [64].

Although the activities that occur during this period trigger behavioral responses [65], primarily, there exist neurophysiological phenomena in which the levels of neuronal plasticity in the offspring and the mother are more or less sensitive to experiences or environmental stimuli, depending on the species [66]. In the mother, the sensitive period is activated with the Fergusson reflex, a signal that is projected to the spinal cord, hypothalamus, and releases oxytocin (OXT) [67]. OXT stimulates uterine contractibility during parturition but also acts on the mother’s olfactory bulb, and allows the secretion of dopamine (DA), thereby causing the mother to identify her offspring [68].

Modifications of neuronal plasticity may involve such mechanisms as synaptic consolidation through molecular adhesion cells, abolition of the activation of synapses because of the insertion of stabilizing molecules, and the construction of synapses through the growth of axons or dendrites. During this period, the influence of neurotransmitters and hormones is important for the establishment of the bond [69], but other factors can also alter mutual recognition at birth.

## 4. Factors That Influence the Bonding Process

The success or failure of dam–offspring bonding depends on the performance of certain behaviors, either by the mother toward her newborn, or vice versa. Primarily, communication pathways include tactile, visual [70,71], olfactory [72,73], and auditory stimuli [74], mainly related to the genetic background of each species and prior maternal experience.

### 4.1. Maternal Care

For proper maternal behavior to be carried out, various hormonal changes occur that trigger acceptance and interactions between the mother and the offspring, activating the attraction–acceptance circuit [75]. Maternal behavior includes feeding and defensive reactions such as increased vocalizations and locomotor activity when separated from their offspring [76]. The mother’s brain is ‘maternalized’ by the hormones that provide emotional rewards for suckling, huddling, and grooming. At this point, the posterior pituitary gland releases (through neurological stimulation) the neuropeptide OXT [77,78,79,80], a nonapeptide that is critical for the neural processing of olfactory and social cues [81], increasing maternal interest in newborns because it helps reduce anxiety and stimulates maternal care [75,82]. This release of oxytocin occurs through two pathways: the Ferguson reflex, which consists of the pressure of the fetal head on the cervix at the time of parturition [83], and the stimulation of the newborns in the mammary gland at the time of suckling [77].

Studies of various mammal species have demonstrated that maternal care participates in the development of social skills, the brain, emotions, and certain behaviors [13]. For instance, in dogs, the amount of maternal care received during early life was associated with different patterns of behavioral responses and the coping strategies of puppies at two months of age [13]. Maternal care was also shown to influence hippocampal plasticity and cognitive functioning in rats, as well as decrease rodent pups’ fear responses [10].

These long-term effects of maternal care on the offspring’s behavior, emotional function, learning, memory, and neuroplasticity are notably mediated by epigenetic mechanisms that influence the transcriptional activity of many genes in the mammalian brain [84]. More precisely, some authors showed that early life experience (i.e., amount of parental care received) affected DNA methylation of oxytocin receptors (OXTR) in the nucleus accumbens of prairie voles offspring, hence altering the OXTR expression in this brain region [85,86]. Moreover, Champagne et al. [87] deciphered the mechanism of OXTR expression involvement in the “transmission of maternal behavior” between the mother and her offspring, which is mediated by the estrogen receptor genes’ epigenetic modification in the MPOA in the hypothalamus; estrogen receptor activation acts to induce oxytocin receptor expression, hence, higher estrogen receptor expression causes higher oxytocin receptor expression. In turn, the oxytocin system in the MPOA is associated with the induction of maternal behavior. Thus, these epigenetic modulations might result in individual differences in maternal behavior [88].

The degree of maternal experience in females is also enhanced in multiparous animals, particularly in precocial species [89]. However, in virgin animals, such as rats, physiological and sensory signaling from their environment can induce maternal behavior. For example, low levels of corticosterone increase licking in virgin rats [90], and this maternal behavior is associated with the cell proliferation of the subventricular zones of the nulliparous rat brain [91]. Although maternal behavior can be induced with the use of hormones, such as estradiol or progesterone, maternal experience also depends on learning during the rearing of the pups, where offspring raised by their mothers become maternal more quickly and have high-licking behavior without the need of external hormones [92].

### 4.2. Importance of Nest-Building in Altricial Species

Nest-building provides shelter and comfort to the newborns and serves as a microclimate refuge station to prevent heat loss [93], to retrieve and transport the newborns there to have protection from predators and lick or nurse the offspring [29,30], particularly in altricial neonates that are born naked and with limited energy resources to thermoregulate [52]. Building the nest is an important action in most primates and rodents. Ungulate dams do not require building a nest since the newborns can actively move after the first hours of life and can respond to vocalizations calling them. Therefore, the predisposition of the mother to nest-building depends on the degree of the physical development of the offspring [24].

In rabbits, mice, and rats, nesting is the center where mother–young interactions take place [27]. For example, rats emerge from the nest by Days 19 and 20 [31], and mice spent at least 50% of their time with the mother in the nest within the first 21 days [94]. Long–Evans rat pups display motor activity to reach and huddle in the central positions of the nest at the first two postnatal days, an effect that is also influenced by the body mass of the animals, where light pups need to compensate for their high mass-to-volume ratio of heat loss [95]. This behavior and burrowing are considered indicators of well-being in rodents [96], but also a way to evaluate the influence of chemical signals that impede maternal care. Regarding this, mice lacking 2 adenylyl cyclase failed to construct a nest and did not retrieve pups during postpartum, which shows the importance of the main olfactory system to detect odorants or pheromones to start maternal behaviors [97].

For newborn rabbits, the nest is essential to survival during the first weeks of life due to the limited assistance of the mother at birth. The doe only feeds the pups once or twice per day for 4 to 5 weeks [98]. The suckling sessions have a duration of about 3 to 4 min. Therefore, the rest of the time the newborns huddle into the insulated nest material to save energy and prevent hypothermia [99].

Other species, such as marsupials, are born in an embryonic stage and immediately after birth reach the pouch and nipple of the mother to fully develop outside the uterus [27]. For these species, the role of a nest can seem less important; however, as Rowland et al. [100] studied in arboreal marsupials, tree hollows, instead of nest boxes, aid in the thermostability of neonate marsupials. According to the results, microclimates inside tree hollows provide a consistent thermal environment with temperatures no higher than 38 °C, in comparison to the nest boxes where the highest recorded temperature was 52 °C, a value that can trigger heat stress in the newborn.

In sows, nest-building is a natural behavior that permits piglets to be protected for long periods (up to seven days post-farrowing) and starts with foraging, rooting, and pawing [93]. This process starts three days before parturition, and studies conducted by Yun et al. [101] have demonstrated that high OXT concentrations (between 7.5 and 23.5 pg/mL) are associated with this behavior before farrowing, and that prolactin (between 23.9 and 27.0 pg/mL) and oxytocin participate in nesting after farrowing, on Days 1 to 7 of lactation. Domestication has altered the presentation of this behavior, and in intensive production systems, the animals are not able to perform it inside farrowing crates [102]. However, if given the required material, such as straw and non-confined environments, sows engage in this pre-farrowing behavior [103].

As has been mentioned, maternal behavior is triggered by the parturition process, the presence of the newborn, and by distinctive neural and endocrine signaling—being tactile, auditory, olfactory, and visual—that promotes the bonding and selective recognition of the filial offspring.

## 5. Neural Pathways Involved in the Bonding Process of Altricial Species

Maternal care and the attachment system in the mother–offspring binomial differ among species and depend on several factors: the newborn’s condition at birth, postnatal development, and numerous neural circuits [104]. The cognitive, sensory, and locomotor capacities of both precocial and altricial species are associated with prenatal neurogenesis and degrees of brain maturation. Consequently, the maternal behavior towards the newborn (altricial and precocial) animals is an important way of promoting the survival of the offspring [14].

At birth, the neonate’s mother is the first social contact and the main source of learning [28,39,104]. In altricial species, communication is practically unidirectional with multimodal signals being sent from the mother to her newborn but few going in the other direction [38].

Mother–young bonding involves structural changes in cortical regions and the continuous release of neurotransmitters [105] that respond to visual, auditory, tactile, and olfactory sensory stimuli [106]. Brain structures, such as the medial preoptic area (MPOA), ventral tegmental area (VTA), medial prefrontal cortex (MPC), and anterior cingulate subregions, dictate maternal motivation in the post-parturition period (on Day 8), while the medial prefrontal cortex prelimbic subregion contributes to late post-parturition maternal care (at day 16) [107].

**Figure 2:**
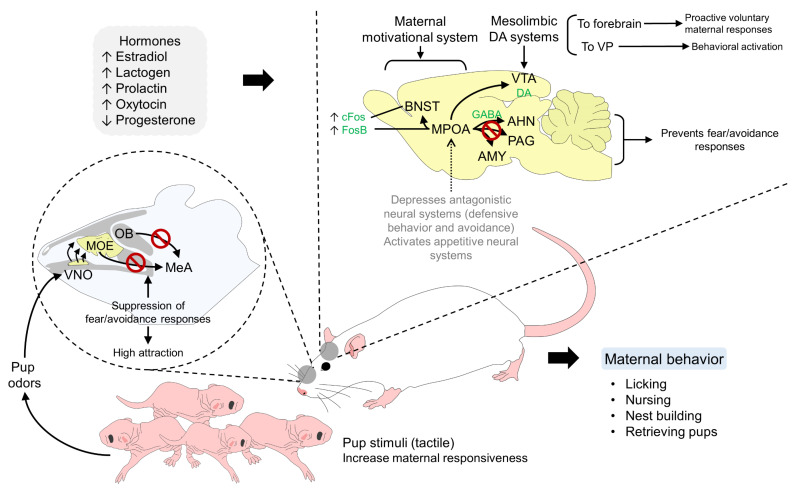
Neurobiology of maternal behavior in altricial species. To develop a maternal response in females, a series of neuroendocrine and sensorial stimuli need a dual interaction in cerebral structures, such as the MPOA, BNST, VTA, AMY, VNO, and OB, among others, to suppress aggressive and avoidance behaviors, while activating appetitive and motivational maternal pathways. AHN: anterior hypothalamic nucleus; BNST: bed nucleus of the stria terminalis; cFos: transcription factor and marker of neuronal activity; DA: dopamine; GABA: gamma-aminobutyric acid; FosB: Fos protein; MeA: medial amygdala; MOE: main olfactory epithelium; MPOA: Medial PreOptic Area; OB: olfactory bulb; PAG: periaqueductal gray; VNO: vomeronasal organ; VP: ventral pallidum.

The neuroendocrine response induces changes in the expression of a range of neuropeptides (e.g., b-endorphin, corticotrophin-releasing factor (CRF), OXT and arginine vasopressin (AVP)), substances that mediate maternal and other social behaviors according to the nature of the received stimuli [15,23]. For example, in the case of rodents, licking, nursing, nest-building, retrieving pups, and feeding the offspring are a group of behavioral responses triggered by a sensorial and endocrine stimulus. It requires the activation of maternal recognition systems and the suppression of responses such as defensive or aversive reactions to the newborn. To achieve this, the hormonal changes involving estradiol, OXT, prolactin, and progesterone during parturition act on specific receptors located in the hypothalamus (MPOA) and the bed nucleus of the stria terminalis (BNST) to activate maternal motivational systems. Simultaneously, the suppressed projections to the amygdala and other structures prevent fear and avoidance behaviors, resulting in proactive voluntary responses to nurse and care for the newborn (Figure 2) [63].

### 5.1. Licking and Tactile Sensitivity of the Brain

Numerous studies have highlighted the importance of licking the newborn during parturition, not only because it provides benefits to the offspring but because it can also reduce the mother’s stress [108,109,110].

In species such as horses, dogs, cats, and rats, among others, the hippocampus was observed while licking the newborn and an increase in glucocorticoid receptors was observed, thus conditioning the functioning of the hypothalamic–pituitary–adrenal (HHA) axis, a key system for the development of an autonomic/endocrine response, unconscious memory, and the regulation of emotional states in the face of external stressors [111,112]. For kittens, the mother is the one who initiates maternal nursing by tactile stimuli to the newborn, such as nuzzling and licking them, to guide them to the nipples to start suckling [64].

During parturition, the rabbit does constantly lick their hind limbs or the genital region of the recently born pup. However, contrary to rats, rabbits spend more time licking the blood, amniotic fluids, or eating the placental membranes than licking the newborn [113]. This reaction is associated with the limited interaction between the doe and the pups, with an average duration of 2 to 4 min [114]. Therefore, in these species, the thermal and olfactory cues emanating from the female are also the main signaling channel of the mother–young recognition [115]. Although it may be limited, rabbit pups can actively crawl and search for the nipple to suckle [114].

### 5.2. Olfactory Stimuli

Olfactory and odor stimuli are some of the most selective pathways for mother–young bonding [116]. The maternal brain undergoes neurogenesis, a type of adaptative plasticity of neurons, especially in olfactory structures, to facilitate olfactory memory [117]. Therefore, interneurons participate in the recognition of the offspring during the sensitive period [118].

Research has shown that non-lactating females are aversive to neonatal odors [119]. However, during postpartum, olfactory cues are partially inhibited to prevent neophobia [120], and these signals are a key stimulus to activate the maternal motivation system [119]. Mothers often recognize their offspring by smell, related to the release of OXT in the brain [121,122,123].

Odor stimuli from pups activate regions in the MPOA, BNST, or the lateral habenula and ventral tegmental area (VTA, through the action of OXT and prolactin [105]. Furthermore, norepinephrine in the brainstem projects on the paraventricular nucleus (PVN) in the hypothalamus and olfactory bulb. In turn, OXT neurons are activated in the PVN. They are responsible for promoting three mechanisms associated with maternal behavior: (1) OXT stimulation of the OB promotes maternal behaviors and reduces aggressiveness towards newborns; (2) Its action on the hypothalamus inhibits postpartum estrus; (3) It promotes behaviors associated with brood care in the VTA [105]. The birthing process triggers the Fergusson reflex through vaginocervical stimulation, increasing the oxytocin concentrations in the PVN.

In the case of rodents, the recognition of newborns and the development of social behaviors mainly depend on the detection of olfactory signals by the dam’s main and accessory olfactory system [15]. In rats, the cortex receives olfactory inputs via the mediodorsal thalamic nucleus, and lesions to this system decrease the frequency of mother protection against cannibalism [124]. The amygdala also participates in the bonding process since lesions in that region alter the process of olfactory preference [125]. Detection of chemical signals induces maternal behavior in virgin females, such as retrieving isolated young [126]. Other studies have discovered that dodecyl propionate (DP), a pheromone released by the preputial gland of rodent pups, is associated with the mother’s licking [9]. Contrarily, Morgan et al. [127] reported that maternal behavior is not affected if the vomeronasal organ is extirped in rats. Moreover, Brouette-Lahlou et al. [128] described that the pups’ survival rate is not related to the vomeronasal activity.

In mice, the removal of the olfactory bulb eliminates maternal aspects in females [129]. A study by Liang et al. [130] concluded that the mother *Tylonycteris pachypus* bats recognize their young by scent. Although pheromones and olfactory stimuli are important for altricial species, in rabbit pups it has been reported that they search for the nipple with or without the association of suckling with odors [131].

### 5.3. Auditory Stimuli

Auditory cues have been described in bird species like penguins [132,133,134] and swallows [135], but are still little studied in mammals. In the case of dog puppies, maternal separation induces vocalizations in newborns from 6 to 8 weeks old, while high maternal care reduces whining and yelping emission, a sign of reduced distress [13]. In cats, vocalization from kittens elicits postural changes (e.g., lactation position) in the mother, and is also an exploratory approach to retrieving the offspring [136].

In rats and mice, pup vocalization highly influences the mother–pup bond. In this sense, the pups’ ultrasonic vocalizations play an ethologically important role in mother–offspring communication by stimulating specific maternal behavioral responses [137]. Pups can emit vocalizations that reach intensities of 30–90 kHz. Ultrasonic vocalizations of rat pups have been recorded at 44 kHz in response to stressful environments such as maternal separation, unfamiliar odors, and thermal or tactile stimulus during birth, and their function is to call the mother to promote retrieval, grooming, and maternal care [138].

Maternal responsiveness can be modulated by the vocalization emitted by pups. For example, D’Amato et al. [139] reported that on the eighth postnatal day, female mice respond to calls from filial pups more frequently than those coming from alien individuals (latency time of 44.3 vs. 93.1 s, respectively). This response is also mediated by prolactin, according to Hashimoto et al. [140]; in 19 lactating rats, an increase in prolactin levels was registered after pup vocalization, eliciting retrieving and nest-building for the newborns. Conversely, in pups, maternal separation alters ultrasonic vocalization, producing low-frequency calls during isolation (around 40 kHz), but high maternal care was associated with more low-frequency vocalizations [141].

As observed in rodents—but also other species—maternal interaction and the responsiveness of the brain to neonatal cues highly depend on the participation of hormones and other neurochemicals that initiate and activate the cerebral structures involved in mother–young bonding.

### 5.4. Visual Stimuli

The cerebral hemispheres and roof of the forebrain are the main sites of visual recognition, as well as the prefrontal and cingulate areas of the cortex in mammals [142].

For canines and felines, the visual system, composed of the retina and axons from the optic nerve that project on the lateral geniculate nucleus, is essential for the perception of the environment [143] and, therefore, to recognize the offspring at birth. Although eyesight is important, in rabbits there was no behavioral difference in maternal care between normal and genetically blind individuals, showing that 98% and 97% of the animals in each group, respectively, developed expected newborn care, suggesting that other signaling pathways, such as auditory and olfaction, can compensate for this impairment [144].

Similarly, in mice, visual impairments due to albinism have not been associated with altered maternal behavior [145]. The same was reported by Sturman-Hulbe [146] in 15 female albino rats, for whom conditions such as blindness and anosmia did not result in altered maternal fitness, and other activities (e.g., nesting, suckling, and cleaning) were similar to those observed in healthy animals.

These findings show that, although visual signaling aids in the development of mother–young bonding, it does not directly trigger maternal behavior since animals require or use other neuroendocrine pathways to respond after parturition.

## 6. The Role of Neurobiological Systems for Mother–Young Recognition and Bonding

### 6.1. Neurotransmitters and Neurohormones

OXT is the main neurohormone associated with social recognition, maternal behavior, parturition, and milk ejection [14,81,88,104,147,148,149]. It is synthesized in the magnocellular neurons of the paraventricular (PVN) and supraoptic (SON) nuclei of the hypothalamus and is projected towards the posterior pituitary for further release into the peripheral circulation. Differences in individual maternal attachment are associated with the evolution of the dopaminergic and oxytocinergic neuroendocrine system, where OXT activates the mesocorticolimbic dopaminergic system in response to social signals. This is crucial for the expression of affiliative behaviors [148,150,151].

The perinatal role of the OXT system in animals is associated with dysfunctional maternal behaviors [152]. For example, in rats, OXT mediates the initiation of maternal behavior [153], even in virgin rats receiving intracerebroventricular doses [154], and OXT knockout mice have a low prevalence of pup retrieving and licking in comparison to nulliparous females [155]. Likewise, the administration of OXT antagonists inhibits maternal behaviors and interferes with the bonding process in the same species [152]. Aggression and maternal defensive behavior on Wistar rats also elicit a higher OXT release in the central nucleus of the amygdala and the PVN [156].

Maternal care such as licking is also related to OXT levels and OXT receptors in the central nervous system, with less expression of these in the hippocampus and OB in dams that lick the pups constantly [157], a trait considered to measure maternal care in this species. Likewise, the same kind of dam has high levels of dopamine (DA) receptors in the nucleus accumbens [158], estrogen receptors in the MPOA, and OXT receptors in the amygdala and the bed nucleus of the stria terminalis [157].

The interaction of OXT with other substances that might be secreted by the organism during the peripartum also dictates the maternal patterns that mothers elicit. In this sense, in rodents, prenatal stress activates the hypothalamic–pituitary–adrenocortical (HPA) axis, decreasing motor development and social and learning skills in the early stages, while OXT in the postpartum period can lower anxiety and depression [159]. It is important to decrease anxiety during the days after parturition [82] so the dam can easily accept the newborn, facilitating social bonding [160]. Specifically, it stimulates maternal behavior in dams and increases maternal care toward the offspring that decreases anxiety [75]. Additionally, Sohlstrom et al. [161] found that exogenous administration of OXT to rat pups in the first days of life results in lower corticosterone concentrations, low blood pressure, and greater weight gain in adults.

Prolactin is another important neuropeptide. Its main role is for milk production and ejections. However, it also participates during maternal care and parental behavior in birds and mammals [162,163,164]. In mice, prolactin promotes neurogenesis in the forebrain and the olfactory bulb, directly participating in the olfactory recognition and acceptance of the newborn [165]. Additionally, it regulates offspring-oriented care not only in females by promoting paternal behavior, shifting the endocrine response from one aimed at fertility to one focused on childcare [166]. Other substances secreted by neurons at this stage and that stimulate the limbic system are gamma-aminobutyric acid (GABA), glutamic acid (GLU), monoamines such as DA and serotonin (5-HT), as well as N-methyl-D-aspartate (NMDA) [167,168].

Additionally, the main function of GABA is to inhibit the signal transmission to cerebral structures such as the amygdala, thalamus, prefrontal cortex, and hippocampus [169], altering the affective aspect through inhibitory GABAergic connections [170]. In the MPOA and BNST, the administration of the GABA receptor agonist in lactating rats causes dose-dependent behavioral deficits such as lack of pup retrieving and maternal aggression, but does not affect licking [171]. In rat pups, catecholamines, such as DA and noradrenaline, have an influence on cardiac, respiratory, and motor activity in the first post-birth days [172]. The release of DA in nulliparous and multiparous female rats when exposed to pup stimuli was studied by Afonso et al. [173]. The researchers found that multiparous females had greater concentrations of DA in the nucleus accumbens, so it can be concluded that maternal experience is mediated by this neurotransmitter.

In the case of 5-HT, authors such as Angoa-Pérez et al. [174] found that female mice with mutations that cause 5-HT depletion have poor maternal performance (e.g., lack of pup retrieving, huddling, nest construction, and high-arched bac nursing), and also affected the survival rate of their offspring. The interaction of 5-HT with nitric oxide (NO) influences aggressive behavior toward the newborn due to diminished 5-HT concentration and lack of NO synthase [175]. Table 2 summarizes the mentioned neurotransmitters and their role in maternal recognition.

Interestingly, these neurohormonal/neurotransmitter parameters can be assessed to investigate the quality and development of mother–young bonding. In this context, the most studied neuromodulator is OXT [87,88,184,185,186]. Unfortunately, the methods used to measure OXT face several pitfalls, notably because there is no methodological consensus about the different OXT assays and pre-analytical treatments used: enzyme-linked immunosorbent assay, radioimmunoassay, liquid chromatography-based methods, with or without pre-analytical treatment steps, such as solid-phase extraction, reduction-alkylation, among others [187,188,189,190,191]. This leads to measurement discrepancies and inconsistent results [189,192,193] and raises the question of the existence of various molecular forms of OXT and their respective biological relevancy [192,194,195]. Additionally, another issue revolves around the matter of the sample volume and availability: OXT levels can be measured in excretory fluids (urine and saliva), blood, or cerebrospinal fluid, these two latter being more invasive but providing more consistent and robust results [189]. However, peripheral measures of OXT may not reflect the central nervous system activity. Cerebrospinal fluid, or even brain microdialysates, are the ideal samples to study the effects of OXT at the central level [196,197], hence, on the regulation of maternal behavior and young bonding. However, the sample collection methods are complicated and often provide only small volumes for the subsequent analyses; this constitutes substantial technical limitations, which could impact the quality of the final measures and the robustness of the findings [197,198].

### 6.2. Hypothalamic–Pituitary–Adrenal (HPA) and Hypothalamic –Pituitary–Thyroid (HPT) Axes

The endocrine response, because of bonding and maternal stimulation by the newborn and their signaling, is largely influenced by the hypothalamic–pituitary–adrenal axis (HPA) and the hypothalamic–pituitary–thyroid (HPT) axis.

The HPA axis is a complex system that regulates the body’s response to stress. It involves interactions between the hypothalamus, pituitary gland, and adrenal glands, which together control the release of stress hormones such as glucocorticoids (GC: cortisol and/or corticosterone). During pregnancy and parturition, the HPA axis is activated, which leads to an increase in GC levels. This increase in glucocorticoids is thought to help the mother adapt to the physical and emotional demands of parturition and motherhood [199]. The activation of the HPA axis and the increase in GC also stimulates the release of OXT, a hormone that is important for maternal bonding and lactation as stated earlier, as a reaction to dampen the HPA axis and downregulate the GC stress response [200]. After parturition, GC levels typically decrease, and OXT levels increase, which is thought to promote maternal bonding and attachment to the young. The HPA axis also plays a role in regulating the mother’s emotional state since it may be affected by dramatic hormonal shifts, which occur during pregnancy, parturition, and the postpartum period and influence her ability to bond with her offspring [201]. Additionally, studies have shown that exposure to stressors during pregnancy may negatively impact the HPA axis, maternal behavior, and mother–young bonding, in link with the oxytocinergic system [201,202]. In turn, maternal behavior serves to “program” HPA responses to stress in the offspring in rodents, the expression of GC receptors in the newborns’ hippocampus is affected by dam licking [19] and pups receiving high tactile stimulation secrete low amounts of corticosterone, representing a least marked stress response [148]. In addition, it has been shown in rodent pups that a hypo-functioning HPA axis is necessary to prevent pups from learning an odor aversion to their mother in the nest, thus allowing the recognition of the mother and the bond formation with her [149].

Overall, the HPA axis is thought to play a role in mother–young recognition and bonding by regulating the release of hormones such as GC and OXT and influencing the young’s brain activation and development.

The hypothalamic–pituitary–thyroid (HPT) axis plays a role in regulating the production and release of thyroid hormones, which exert broad effects on development and physiology. The two main hormones secreted by the thyroid gland are thyroxine (T4) and 3,5,3′-triiodothyronine (T3). While thyroid hormone status is associated with mood dis-orders, limited information is available about their involvement in social behaviors, including maternal behavior [203]. However, several studies have shown that thyroid hormones are implicated in the regulation of OXT and its signaling, acting on OXT plasma levels and OXT receptors’ transcriptional regulation [204,205,206]. Hence, via their impact on OXT, maternal thyroid hormones could play a part in maternal behavior development and the establishment of mother–young bonds. Additionally, Stohn et al. [203] showed that an abnormal local regulation of thyroid hormone action in the mice brain due to type 3 deiodinase deficiency is related to abnormalities in the OXT system (low adult serum levels of OXT and an abnormal expression of the OXT gene and its receptor in the neonatal and adult hypothalamus). In addition, research on rodents has shown that maternal thyroid hormones influence the development of the brain regions that are necessary for offspring recognition, maternal behavior, and bonding, like the neurogenesis process in the maternal hippocampus [207,208].

Finally, it is noteworthy that the link between the HPT axis and mother–young recognition and bonding is not totally understood; more research is necessary to fully understand the relationship.

### 6.3. Brain–Gut–Microbiome Axis

The brain–gut axis refers to the communication pathway between the brain and the digestive system. This pathway is bidirectional, meaning that signals can travel from the brain to the gut, and from the gut to the brain. Additionally, the gut microbiome, which is the collection of microorganisms that live in the gut, also plays a part in the biochemical signaling events that take place between the gastrointestinal tract and the central nervous system [209,210].

Through the activation of the HPA axis, stress occurring at an early age and involving the bonding with the mother can impact the gut microbiota and the brain–gut axis function of the offspring [209,211]. It was shown that the intestinal permeability and the development of the HPA axis in the offspring were altered by limited nesting stress in Sprague Dawley rats [212]. In the same species, prevention of weaning results in changes in gut health and microbiome [213]. Early life stressful events such as maternal separation have also been implicated in the alteration of the intestinal microflora and the presentation of adult life disorders in the offspring (e.g., irritable bowel disease) [214,215].

The link between the gut–brain axis and the development of bonding between a mother and her offspring is also thought to be related to the role of certain hormones and neurotransmitters in both the gut and the brain. Indeed, the gut microbiome has been shown to play a role in regulating the production of certain neurotransmitters, such as serotonin, that are involved in maternal behavior [181,182,216]. These neurotransmitters and neurohormones can affect the mother’s ability to bond with her child (see Table 2). Additionally, the mother’s gut microbiome can influence her emotional state, which can benefit the bond development between the mother and her offspring [217,218].

## 7. Factors That Affect Maternal Recognition and Performance

**Figure 3:**
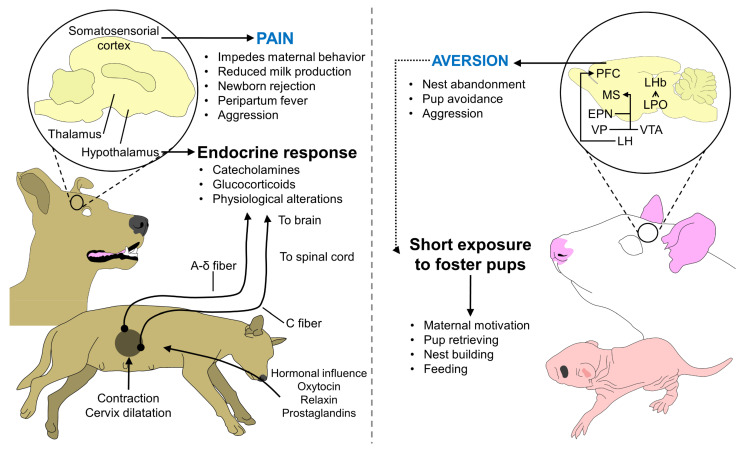
Influence of pain and aversion on maternal behavior in altricial species. Although pain is a physiological trait in the parturition process, if dystocia occurs and the pain is prolonged in the mother, the activation of nociceptors (A-delta and C fibers) activates the sympathetic nervous system, and the consequent release of catecholamines and glucocorticoids. Moreover, the conscious recognition of pain in the somatosensorial cortex alters maternal behavior and reduces milk production, resulting in a negative trait for both the mother and the offspring. On the other hand, the activation of aversion pathways in non-parturient females prevents them from maternal responsiveness. However, in species such as rodents, short periods of pup exposure elicit similar responses in multiparous, parturient, and lactating animals. EPN: entopeduncular nucleus; LH: lateral hypothalamus; LHb: lateral habenula; LPO: lateral preoptic area; MS: medial septal nucleus; PFC: prefrontal cortex; VP: ventral pallidum; VTA: ventral tegmental area.

The acceptance of the newborn in the first hours of life and the activation of the aforementioned neural pathways can be interrupted by a series of events that impede bonding. These events can be included in physiological and behavioral aspects. Regarding physiological elements, parturition pain is part of the normal process due to cervical dilatation and the activation of uterine pain receptors [219] (Figure 3). However, it is known to have a detrimental effect on the degree of maternal behavior [220], particularly during dystocia [221]. This has been reported in semi-altricial species such as pigs, whose peripartum pain causes consequences such as reduced food intake (hyporexia), reduced milk letdown, a lower ability to care for the newborn [222,223], and the use of anti-inflammatory drugs can decrease the presentation of peripartum fever, a disorder present in 40 to 100% of farrowing with a duration of 4 to 8 h [224]. For example, Kuller et al. [222] showed that pain and fever relief using paracetamol during peripartum improved the performance of the mother and reduced the variation of weight within litters and back fat loss during lactation. Pain can also be accompanied by cortisol and tumor necrosis factor α increase in multiparous sows, a stress-related response [223]. The use of analgesics has also shown a beneficial effect on mastitis-metritis-agalactia syndrome in sows, where Hirsch et al. [225] found that meloxicam and flunixin have similar efficiency when considering rectal temperature, inflammation of mammary glands, milk letdown, and nursing behavior (*p* < 0.05), but lower mortality rates were observed in litters from meloxicam group.

Another factor that can result in nest abandonment and even cannibalism is nervousness and fear-related responses in altricial species. Contrary to pregnant animals, whose fear and defensive behaviors are suppressed due to hormonal stimulation [226], in nulliparous or virgin rodents, avoidance is the first reaction to pups when exposed to newborns, and timidity and fearfulness are traits present in these animals [227]. However, maternal motivation can be induced in virgin rats and mice after only 5 to 21 days of exposure to pups [228,229], and even within the first 15 to 30 min [230]. The induced responsiveness, similar to parturient females retrieving pups and crouching over the offspring, shows that females can activate social mother–young bonding pathways even with foster offspring [88].

Nonetheless, when aversion to neonatal stimuli persists, nest abandonment and neonate mortality by a lack of nursing are observed in species such as altricial birds [231]. In them, the presence of predators such as mice can cause an up to a 10-fold increase in the rate of nest abandonment and, therefore, consequences to offspring survival [232]. In the case of mammals, together with the abandonment of the offspring, events such as cannibalism have been reported. Altered maternal responses such as cannibalism and rejection of puppies can be found in at least 10.7% of breeders [233]. In Kangal dogs, several studies reported maternal cannibalism occurring during the first 24 h after birth, and an association with high levels of adenosine deaminase and xanthine oxidase, metabolic enzymes that can be studied since cannibalism is associated with nutritional deficiencies and environmental stressors [234].

Regarding the nutritional state of the mother before and during parturition, low lipid levels (high-density and low-density lipoprotein, as well as cholesterol) were found by Kockaya et al. [235] in Kangal dogs with a history of cannibalism (*p* < 0.05), as well as low OXT concentration. However, cannibalism is not only related to a detrimental mother–young interaction. It can also be caused by the high population density of a species within an area, as reported in dingoes in Australia [236].

Moreover, human–animal interaction can potentially alter the degree of acceptance of the newborn by the mother, as well as the social behavior and human-related response of the offspring when reaching adulthood [237]. Additionally, it is relevant to mention that performing ethological studies regarding maternal behavior requires special attention to sample size and reliable methods to obtain robust results. For example, it has been observed in ewes that human intervention alters the parturition process and, consequently, could affect the bonding period with bad-quality maternal care [238]. Therefore, the aspects that need to be considered during parturition and the sensitive period are the physiological and emotional states of the mother, as well as the intervention in the process.

## 8. Future Directions

Understanding the neurobiological pathways in mothers during the bonding process can help to avoid situations that cause discomfort or anxiety in females, and thereby improve their productivity. Many studies showed a correlation between the anxiety of primiparous mothers and the amount of maternal care in mammals [239,240,241], including dogs [242]. In the same way, by improving the facilities so that there is no stress on the mothers and the offspring, the imprinting process will develop without failure.

An important factor is recognizing the importance of nesting behavior in altricial species in commercial breeding units. In some cases, such as with fattening rabbits, not providing the mother with material to nest could affect the survival of the newborns, making them vulnerable to environmental factors [99]. To assess these behaviors, more studies focusing on precision livestock farming, such as Oczak et al.’s [243] work, could help evaluate the presence of this behavior with a sensitivity of 87% and specificity of 85%, an alternative that can be applied to other species to improve their welfare and the newborn survival.

Likewise, knowing the interaction between neurochemicals, brain structures, and behavior during and after parturition could help to propose protocols in cases of maternal cannibalism in species such as dogs [244] or piglets. This could be an approach that would also represent an advantage for the newborn and future gestation periods.

## 9. Conclusions

Bonding is an essential process for dams and newborns, where several factors play a key role in its development. Inherent characteristics of the animals such as the type of species (e.g., altricial and precocial) contribute to the degree of maternal care that the newborn requires during the first days of life. For example, altricial offspring born with limited motor capacities and immature sensorial systems often require prolonged nursing inside a nest or near the mother to obtain energy resources and protection.

The signaling of this interaction is triggered by brain pathways activated by external stimuli and neurotransmitters and hormones present at parturition. The presence of the newborn, their odor, vocalization, and tactile stimuli are processed by brain structures such as the MPOA, amygdala, visual and auditory cortex, as well as the olfactory main system, causing the release of dopamine, OXT, and prolactin, among others, to develop maternal motivation and a selective bond with the offspring.

A mother–young recognition represents a beneficial trait for the newborn and its survival, and contributes to the maternal experience for future litters. Thus, considering the necessary elements for this process to start (e.g., a sensitive period) is a way of avoiding elements that may interfere with bonding. Particularly, in productive units, strategies aimed to protect this period are relevant in the improvement of the welfare and commercial value of the animals, preventing neonatal losses, and gaining mothers with previous experience to raise their offspring.

## Figures and Tables

**Table 1:** Summary and comparison of maternal bonding in altricial and precocial species.

Species	Pre-Natal Development	PostNatal Development	Functional Senses Immediately after Birth	Maternal Care	Bonding	Reference
Altricial	Fetal response to umbilical cord occlusion	Lung Brown adipose tissue Brain Muscle	Limited locomotion Hairless Limited thermoregulatory ability Unable to hear and see immediately after birth	Require nesting Require constant parental care	Nursing period within the nest Mother–infant bonding in the first postnatal weeks	[49,50,51]
Precocial	Organogenesis in utero (lung, liver, and brain) Muscular development Piloerection Brown adipose tissue	Coordinated locomotion	Sight, hearing Fully covered in fur Locomotor capacity	Do not require constant care. Do not need nesting	Bonding in the first postnatal hours	[52,53,54,55]

**Table 2:** Main neurotransmitters involved in mother–young recognition.

Neurotransmitter	Synthesis	Status	Role	Reference
OXT	PVN, SOP	↑	Maternal behavior	[153]
		↓	Lack of newborn retrieving and licking.	[155]
GABA	Presynaptic neuron	↑	Maternal defense	[176]
GLU	Presynaptic neuron	↑	Long-term maternal experience.	[177]
DA	Dopaminergic neurons	↑	Maternal care, bonding, reward system.	[178]
		↓	Impaired maternal recognition.	
PRL	Lactotrophs in the anterior pituitary gland	↑	Maternal care.	[179]
		↓	Litter abandonment.	[180]
5-HT	Enteric nervous system, CNS, Merkel and pulmonary cells	↑	Modulates DA maternal effects.	[181]
		↓	Reduces pup survival and nursing behavior.	[182]
NMDA	Ionotropic neurons	↓	Impaired retrieval.	[183]

5-HT: serotonin; CNS: central nervous system; DA: dopamine; GABA: gamma-aminobutyric acid; GLU: glutamate; NMDA: N-methyl-D-aspartate; PVN: paraventricular nuclei; SON: supraoptic nuclei.

## Data Availability

Not applicable.
